# Occurrence of *Panstrongylus megistus* (Burmeister, 1835) in an area under entomological surveillance in the Southeast Region of Brazil

**DOI:** 10.1590/0037-8682-0084-2020

**Published:** 2020-12-21

**Authors:** João Victor Leite Dias, Rita de Cássia Moreira de Souza, Janice Maria Borba Souza, Liléia Gonçalves Diotaiuti, Raquel Aparecida Ferreira

**Affiliations:** 1 Universidade Federal dos Vales do Jequitinhonha e Mucuri, Campus Teófilo Otoni, Faculdade de Medicina do Mucuri, Teófilo Otoni, MG, Brasil.; 2 Instituto René Rachou, Grupo de Pesquisa Triatomíneos, Belo Horizonte, MG, Brasil.; 3 Superintendência Regional de saúde de Divinópolis, Divinópolis, MG, Brasil.

**Keywords:** Chagas disease, Surveillance, Triatomine

## Abstract

**INTRODUCTION:**

*Panstrongylus megistus* is the main triatomine involved in the human transmission of *Trypanosoma cruzi* in Minas Gerais, Brazil. We analyzed the occurrence of triatomines in the Itaúna micro-regions for healthcare.

**METHODS:**

Data were collected as part of routine entomological surveillance activities, including the species identity, capture site, developmental stage, and trypanosome infection.

**RESULTS:**

In total, 503 specimens from five species were captured (495 *P. megistus*). Adults were mainly captured by residents inside their homes, whereas nymphs were mostly captured by public health professionals outside.

**CONCLUSIONS:**

The epidemiologically important triatomine, *P. megistus,* continues to persist in our study region.

Triatomines (Hemiptera: Reduviidae) are important for public health because some species might play a role in the transmission of *Trypanosoma cruzi* ^1^, the causative agent of Chagas disease. Among over 150 described triatomine species, the most epidemiologically important are those able to colonize human dwellings and their associated outbuildings^2^.

In Brazil, the transmission of *T. cruzi* by *Triatoma infestans* (Klug, 1834), a non-native vector, has been effectively interrupted, but native triatomine species remain a target for Chagas disease control programs^3^. In Minas Gerais, the native triatomine *Panstrongylus megistus* (Burmeister, 1835) was the main domiciliary species before the introduction and spread of *T. infestans* during the early 20^th^ century^4^. *Panstrongylus megistus* is currently one of the most frequently captured triatomines in Minas Gerais, and its infestation of houses has persisted, although at low densities. However, the rates of natural *T. cruzi* infection are usually high, and the trypanosome is frequently found in nymph-form inside houses, where they often feed on the blood of humans^5^.

The mid-western region of Minas Gerais has been important for studies on Chagas disease epidemiology, both historically and in the present day, due to the high incidence of the disease in the past and the continuing presence of triatomines, *P. megistus*, the most important species in the region from an epidemiological point-of-view^6^ ^-^ ^7^. The mid-western region comprises 54 municipalities coordinated by the Regional Health Management of Divinópolis (RHMD). In turn, each of these municipalities is grouped into one of six healthcare "micro-regions" to comply with the legislation provided for in the Health Regionalization Plan of Minas Gerais^8^. These healthcare micro-regions are administrative divisions that coordinate the health services offered to an area, providing secondary and, eventually, tertiary healthcare assistance^8^.

This study aimed to describe the occurrence of triatomines in "domiciliary units" (DU) (i.e., the intradomicile and peridomicile of residential buildings considered together) located within the micro-region of healthcare of Itaúna (MHI), which comprises four municipalities (Itaguara, Itatiaiuçu, Itaúna, and Piracema; Figure 1) and is the smallest micro-region under the management of the RHMD. These four municipalities are located 95, 72, 76, and 120 km, respectively, from the state capital (Belo Horizonte), with proximity to this urban area being the main criterion for choosing the MHI for our study. Although they are distinct administrative and political units, the municipalities of Itaguara and Itatiaiuçu are both located in the metropolitan area of Belo Horizonte. In 2015, the MHI had an estimated population of 123,570 inhabitants, residing in an area of 1482.1 km² ^9^, and is located in the Cerrado biome of Brazil.

**FIGURE 1:**
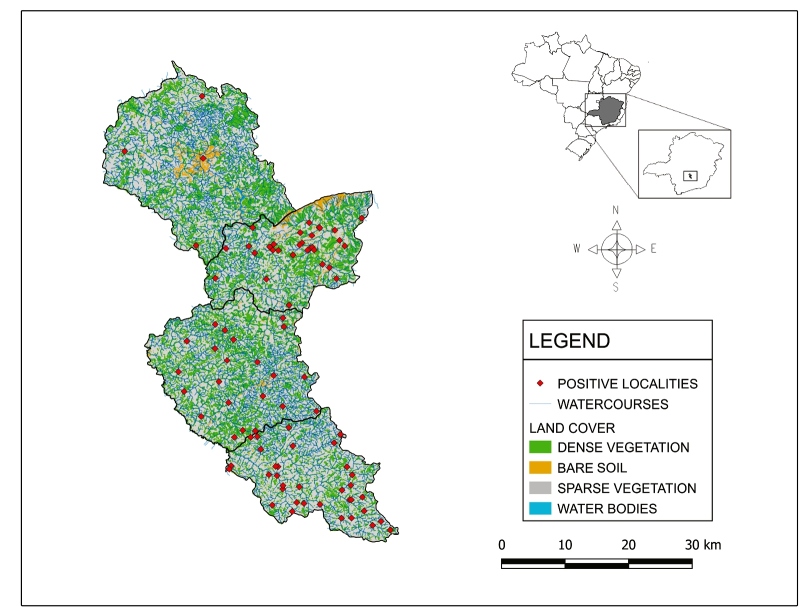
The four municipalities of the micro-region of health of Itaúna (MHI), Minas Gerais, Brazil. From top to bottom: Itaúna, Itatiaiuçu, Itaguara, and Piracema. The red dots represent the localities where triatomines were found. Land cover classes were retrieved from the mean normalized difference vegetation index, calculated with four images from LANDSAT 5 TM (1987, 1997, 2007, and 2011). The watercourses layer was obtained from the Brazilian Institute of Geography and Statistics (IBGE).

Data from the Chagas Disease Control Program "SisPCDCh" between 2011 and 2015, provided by the Minas Gerais Secretary of Health (SES-MG), was used to prepare this report. The SES-MG is responsible for collating and managing all the information on triatomines captured in DU as part of routine entomological surveillance, organized within each healthcare micro-region.

Triatomines were collected in each municipality by local public health agents, who visited human dwellings either in response to residents' notifications regarding suspected triatomines or to perform prescheduled active bug-searches of all DU within a given area. The collected insects were morphologically identified and checked for *T. cruzi* infection, using optical microscopy of the hindgut contents in local laboratories by trained personnel.

Information contained in the SisPCDCh database included the locality where the insects were captured, the species identification of the collected triatomines, the nature of the capture site (whether intradomicile or peridomicile), the developmental stage of the collected triatomine, their positivity for trypanosomatids, and the surveillance mode that led to the capture of the insect (i.e., whether through resident notification or area-wide prescheduled active bug-searches). The association between categorical variables was analyzed using the chi-square test implemented in BioEstat version 5.3^10^ using α = 0.05 as the cut-off for significance.

Additionally, to describe the spatial distribution of triatomine occurrence, the geographic coordinates of infested localities were obtained from the Agricultural Census database for 2006 to 2017, provided by the Brazilian Institute of Geography and Statistics (IBGE). Each locality was represented by a single georeferenced point located at its centroid.

Over the five-year period of our study, 503 triatomines from five species were captured inside or near dwellings (Table 1). *Panstrongylus megistus* was the most frequently collected species (98.4% of all insects), and it was the only species infected with *T. cruzi* (*n* = 5, which included two adults found indoors and one adult and two nymphs collected in the peridomicile). The infected *P. megistus* comprised 1.3% (*n* = 390) of all triatomines examined for *T. cruzi*. These insects were caught in five different DU located in two different localities in the municipality of Piracema and one locality in the municipality of Itatiaiuçu. We were not able to confirm the reason why many of the captured triatomines were not examined for trypanosomes, but, in general, an examination is not performed when the captured triatomines are dead and/or a long time has elapsed between capture and when the examination is performed, such that the hindgut content is no longer available.

Adult specimens (*n* = 288 in total) were mostly captured indoors and by residents (42.4% of all adults; *n* = 122), whereas nymphs (*n* = 215 in total) were significantly more likely to be caught in outbuildings by health workers (73.5% of all nymphs; *n* = 158; Table 1). There were significant associations between the developmental stage of the triatomine and their capture site (χ² = 66.3; *P* < 0.0001), developmental stage, and mode of surveillance (i.e., resident notification versus prescheduled active search; (χ² = 144.2; *P* < 0.0001) as well as the capture site and prescheduled active search (χ² = 85.8; *P* < 0.0001).

**TABLE 1:** The number of triatomines captured in domiciliary environments in the four municipalities of the micro-region of healthcare of Itaúna (MHI), Minas Gerais, Brazil, according to species, developmental stage, capture site (intradomicile or peridomicile), and mode of surveillance (resident notification or prescheduled active search) between 2011 and 2015.

Species	Resident notification				Prescheduled active search				Total	Examined	Positive
	Intradomicile		Peridomicile		Intradomicile		Peridomicile				
	Adults	Nymphs	Adults	Nymphs	Adults	Nymphs	Adults	Nymphs			
*P. megistus*	116	2	39	9	47	46	78	158	495	389	5^‡^
*P. diasi*	4	-	1	-	-	-	-	-	5	-	-
*R. neglectus*	1	-	-	-	-	-	-	-	1	1	-
*T. circummaculata* ^*†*^	1	-	-	-	-	-	-	-	1	-	-
*T. sordida*	-	-	1	-	-	-	-	-	1	-	-
Total	122	2	41	9	47	46	78	158	503	390	5

^*†*^ Specimen misidentified, as this species is restricted to the state of Rio Grande do Sul, Brazil. ^‡^ Two adults in intradomicile (resident notification), one adult (resident notification), and two nymphs in peridomicile (notification and active search).

Overall, from a total of 423 localities within the MHI, 114 (26.9%) reported the occurrence of at least one triatomine between 2011 and 2015, either through resident notification or prescheduled active searches. Among the subset of localities where prescheduled active searches by public health agents were performed (*n* = 619 searches, as some localities were searched multiple times), triatomines were found in 253 (40.9%) of the opportunities (Figure 2). Concerning individual municipalities, 236 (46.9%) of all triatomines from the MHI were captured from 133 DU in Piracema, 175 (34.8%) from 97 DU in Itatiaiuçu, 79 (15.7%) from 37 DU in Itaguara, and 13 (2.6%) from 6 DU in Itaúna. The municipality of Itaúna did not have prescheduled active searches in 2011, 2012, and 2013. In addition, not all insects captured in DU as part of routine entomological surveillance are reported in the SisPCDCh database, as usually only 10%-20%, or even less, of the sampled insects are recorded. Thus, the number of insects captured during the period of our study, 503, very likely represents only a small proportion of the actual number of triatomines collected in DU, and an even smaller proportion of the triatomines actually present.

**FIGURE 2:**
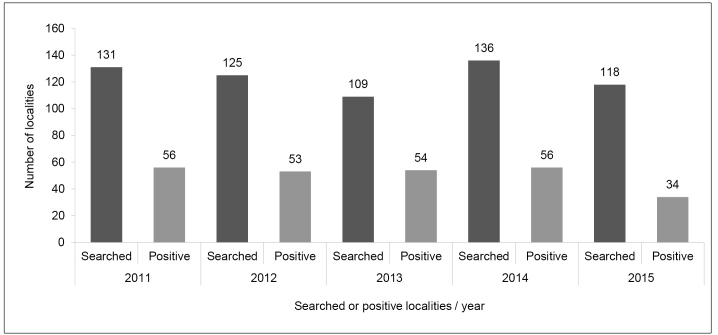
The number of localities searched, and the number of them positive for the presence of triatomines by year in the micro-region of health of Itaúna (MHI), Minas Gerais, Brazil (*n* = 423).

*Panstrongylus megistus* was the most captured species of triatomine. This vector has been captured in many regions of the state of Minas Gerais, where it has colonized domiciles and outbuildings^6^ ^,^ ^11^ ^,^ ^12^. This species was also found to have colonized the intradomicile, and other human-made structures in the municipalities studied. The presence of this species in domiciles may represent a risk for *T. cruzi* transmission to the human residents, as the latter may act as a source of food (blood)^5^. Besides, residents living in endemic areas often find it difficult to identify and detect nymphs^13^ ^-^ ^14^, which is likely to contribute to the persistent presence of immature forms of triatomines inside houses, especially as they were detected mostly by public health professionals.

The widespread distribution of *P. megistus* observed in our study region could be explained by the vast number of watercourses and riparian forests (Figure 1), as they are known to be the preferred habitats for this species, especially within the Cerrado biome. These types of landscape features and vegetation are remnants of tropical Atlantic Forest, which has been hypothesized as the original native habitat of *P. megistus* and from which it dispersed into the Cerrado^4^.

Other species found indoors were *Panstrongylus diasi* Pinto & Lent, 1946, *Rhodnius neglectus* Lent, 1954, *Triatoma circummaculata* (Stål, 1859)*,* and *Triatoma sordida* (Stål, 1859). Except for *T. sordida*, these species are not often observed colonizing domiciles, and thus they are believed to have little epidemiological importance with regard to the human transmission of Chagas disease. It is worth mentioning that the report of *T. circummaculata* is probably an error created during the entry of the species identification code into the relevant field of the SisPCDCh database. The incorrect morphological identification of this specimen is thought to be less likely, as the distribution of this species (*T. circummaculata*) in Brazil is restricted to the state of Rio Grande do Sul^15^. This situation reinforces the need for better communication and feedback between local health professionals and centralized reference laboratories to enable the correct reporting of triatomine occurrence, such as supporting the surveillance services in each municipality with educational and training activities provided by the reference laboratories.

The Brazilian Chagas Disease Control Program was carried out nationwide since the late 1970s, under the responsibility of federal health agents. However, since the early 2000s, municipalities became responsible for maintaining triatomine surveillance and control activities. However, many municipalities stopped entomological surveillance at this time, and data gathering regarding triatomine occurrence was discontinued^6^. In MHI, triatomines are detected when surveillance occurs. In areas where active entomological surveillance is still common, the local community is aware of this and know how to recognize triatomines better than in areas where active surveillance is now rare or non-existent^14^.

 Triatomines were found in a number of the searched localities, with no evidence of a decreasing trend over time. This scenario will probably remain unaltered in the future, as riparian vegetation remnants constitute permanently preserved areas and may act as sources of triatomines for the surrounding houses. The occurrence of *P. megistus* within undisturbed native forests has long been recognized^4^ ^.^ However, given the current importance of preserving biodiversity and native habitats, the destruction of protected forest fragments is not an acceptable way to control this vector of Chagas disease. On the other hand, the continuous presence of anthropophilic triatomines such as *P. megistus* in domiciles of the MHI, including the presence of insects infected with *T. cruzi*, reinforces the need to strengthen entomological surveillance activities in this and other regions, together with intense health education programs for residents, and the continued involvement of public health agents and policy decision-makers.
